# Trends in Alzheimer's disease and heart failure-related mortality among older American adults: Insights from the CDC WONDER database

**DOI:** 10.1016/j.ahjo.2025.100677

**Published:** 2025-11-14

**Authors:** Yasmeen Shaikh, Syeda Shahnoor, Muhammad Ahmed Ali Fahim, Abdul Moiz Khan, Tasleem Shaikh, Abdul Moeed, Muhammad Sohaib Asghar

**Affiliations:** a Department of Internal Medicine, Dow University of Health Sciences, Karachi, Pakistan; b Department of Internal Medicine, Ayub Medical College, Abbottabad, Pakistan; c Department of Internal Medicine, Ziauddin Medical College, Karachi, Pakistan; d Department of Internal Medicine, AdventHealth Sebring, FL, USA

**Keywords:** Alzheimer's disease, Heart Failure, CDC WONDER

## Abstract

**Introduction:**

Alzheimer's disease is one of the leading causes of death among the elderly in the United States with heart failure sharing similar risk factors. This study investigated trends and disparities in Alzheimer's disease mortality among older adults with heart failure from 1999 to 2020 in the United States.

**Methods:**

Making use of ICD-10 codes death certificate data from the Centers for Disease Control and Prevention Wide-Ranging OnLine Data for Epidemiologic Research database was retrieved for patients aged ≥65 years between 1999 and 2020. Age-adjusted mortality rates (AAMRs), per 100,000 people, and Annual Percentage Change (APCs) with their respective 95 % Confidence Intervals (CI) were also calculated. Data was stratified by year, gender, race and geographical distribution.

**Results:**

Alzheimer's disease with coexisting heart failure was responsible for 192,459 deaths between 1999 and 2020. Overall the AAMR increased from 21.32 in 1999 to 24.56 in 2005 (APC: 1.9760*; 95 % CI: 0.6001 to 3.9507) after which a significant decrease to 16.52 by 2013 was observed (APC: −4.9301*; 95 % CI: −6.5209 to −4.0119). AAMRs decreased from this point forward reaching 22.21 in 2020 (APC: 4.1573*; 95 % CI: 3.0373 to 5.7232). Women had higher AAMRs than men (21.57 vs 18.41). Among racial groups, the Non-Hispanic (NH) White (21.62) population had the highest AAMRs followed by NH Black/African American (17.87), Hispanic/Latino (14.3) and NH Asian/Pacific Islander (8.96). Furthermore, AAMRs also varied by census region (West: 24.05; Midwest: 22.83; South: 21.1; Northeast: 13.38). Moreover, nonmetropolitan areas had higher AAMRs compared to metropolitan areas (27.23 vs 19.09). States in the top 90th percentile such as Kentucky, Oklahoma, Washington, North Dakota and Mississippi had AAMRs that were three times higher relative to states in the lower 10th percentile including Nevada, Florida, New York, District of Columbia and Hawaii.

**Conclusion:**

Alzheimer's disease mortality with associated heart failure has shown considerable variation in adults ≥65 years. AAMRs were highest in women, NH Whites, residents of the West and nonmetropolitan patient populations. Targeted interventions and a more holistic approach to patient management are essential in achieving favorable outcomes for vulnerable groups moving forward.

## 1 Introduction

Alzheimer's disease (AD) is a progressive neurodegenerative disorder and the leading cause of dementia, affecting an estimated 6.7 million Americans aged 65 and older as of 2023 [1]. It accounts for approximately 120,000 deaths annually, making it the sixth leading cause of death among older adults in the United States (US) [2]. Heart failure (HF) is a chronic condition characterized by the heart's reduced ability to effectively circulate blood. Currently, an estimated 6.7 million Americans aged 20 and older are living with HF, and this number is projected to increase to 8.5 million by 2030 [3]. The coexistence of AD and HF presents a major public health concern, as these conditions disproportionately affect aging populations and significantly contribute to morbidity, mortality, and healthcare expenditures [4].

The prevalence of both AD and HF has risen over the past two decades due to an aging population and improved diagnostic capabilities [5,6]. Despite advancements in medical therapies, mortality rates associated with these conditions remain high. Research indicates that individuals with coexisting AD and HF face a two- to threefold higher risk of mortality compared to those with either condition alone [4]. Moreover, despite recorded racial, ethnic, and regional disparities, comprehensive analyses of long-term mortality trends among individuals with both conditions remain limited.

This study aims to analyze the temporal trends in AD and HF-related mortality among older American adults from 1999 to 2020 using data from the Centers for Disease Control and Prevention's Wide-ranging Online Data for Epidemiologic Research (CDC WONDER) database. By examining demographic variations and mortality patterns, this research seeks to provide insights into the evolving burden of AD and HF-related deaths, informing public health policies and targeted interventions.

## 2 Methods

### 2.1 Study setting and population

This study utilized mortality data from the Centers for Disease Control and Prevention's (CDC) Wide-Ranging Online Data for Epidemiologic Research (WONDER) database [7]. We examined trends in mortality among individuals aged 65 years and older where AD and HF were recorded as either the underlying or contributing cause of death on U.S. death certificates between 1999 and 2020. Data were extracted from the Multiple Cause of Death Public files of the CDC WONDER database, a widely used resource for analyzing mortality patterns in which a single death certificate may have up to 20 listed diagnoses as causes of death [8].

Cases were identified using the International Statistical Classification of Diseases and Related Health Problems, 10th Revision (ICD-10) codes G30.0, G30.1, G30.8, G30.9 for AD, I50.0, I50.1, I50.9 for HF and I25.5 for Ischemic Cardiomyopathy (ICM). These classification codes have been employed in prior research investigating mortality linked to these conditions [9]. Since the dataset is publicly available, de-identified, and government-provided, institutional review board approval was not necessary. This study was conducted following the Strengthening the Reporting of Observational Studies in Epidemiology (STROBE) guidelines [10].

### 2.2 Data abstraction

This study categorized mortality data based on several demographic variables, including year, gender, place of death, racial and ethnic background, geographic region, and urbanization level. Racial and ethnic groups were classified as Hispanic or Latino, non-Hispanic (NH) White, NH Black/African American, and NH Asian/Pacific Islander, following the standard categorization used in previous CDC WONDER analyses and aligned with the US Office of Budget and Management Guidelines [11]. Places of death included medical facilities (inpatient, outpatient, emergency room, death on arrival or status unknown), homes, hospices and nursing homes.

Additionally, the United States was segmented into four regions—Northeast, Midwest, South, and West—following the classification system established by the US Census Bureau [12]. The study also incorporated geographic categorization based on the Urban-Rural Classification Scheme established by the National Center for Health Statistics, where metropolitan areas included large metropolitan areas (1 million or more) and medium/small metropolitan areas (50,000 to 999,999), while nonmetropolitan areas included regions with fewer than 50,000 people, as per the 2013 U.S. Census [13].

### 2.3 Statistical analysis

This study examined trends in mortality by calculating both crude and age-adjusted mortality rates (AAMR) per 100,000 individuals along with their respective 95 % Confidence Intervals (CI). AAMRs were standardized to the 2000 U.S. population following established methodologies [14]. Pairwise comparisons were performed using z-statistics, where the difference between two AAMRs was divided by the standard error of the difference, with standard errors being calculated from 95 % CIs. Two-sided *p*-values were reported, with statistical significance set at *p* < 0.05. To evaluate temporal trends in mortality related to Alzheimer's disease and heart failure, we utilized the Join Point Regression Program (Version 5.0.2, National Cancer Institute) [15]. This method involved fitting log-linear regression models to assess annual percentage change (APC) and its corresponding 95 % confidence interval (CI). A trend was classified as increasing or decreasing if the slope significantly differed from zero, with statistical significance set at *p* ≤ 0.05 using a two-tailed *t*-test.

## 3 Results

From 1999 to 2020, a total of 192,459 deaths were recorded in the United States, where Alzheimer's disease, alongside coexisting heart failure, was identified as either the primary or a contributing cause of death. (Supplementary Table 1). An autopsy was performed on 464 (0.24 %) of patients, with 77.43 % of patients having no autopsy performed. Autopsy status was unknown for 22.33 % of patients. (Supplementary Table 2) 68.03 % of deaths were female, with 31.97 % being male. Moreover, the highest proportional mortality belonged to NH Whites (87.57 %), residents of the South (35.65 %), and residents of metropolitan areas (76.31 %). When assessing place of death, nursing homes or long-term care facilities (*n* = 108,758; 56.51 %) accounted for the highest number of fatalities, followed by decedents' homes (*n* = 36,264; 18.84 %) and medical facilities (*n* = 30,272; 15.73 %), uncategorized locations (n = 10,932; 5.68 %), and hospice facilities (*n* = 5775; 3.00 %). Place of death remained unknown for a minority of decedents (*n* = 458; 0.24 %). (Supplementary Table 3). Demographic data is provided in Table 1

**Table 1:** Demographic characteristics of deaths due to Alzheimer's disease and heart failure in older adults in the United States, 1999 to 2020.

Variable	Alzheimer's disease and heart failure Deaths n (%)	AAMRs (95 % CI) per 100,000	P-value of Pairwise Comparisons
Overall Population	192,459 (100)	20.56 (20.47–20.65)	
Sex			P < 0.001
Male	61,546 (31.97)	18.41 (18.26–18.56)	
Female	130,913 (68.03)	21.57 (21.46–21.69)	
Census Region			P < 0.001
Northeast	26,505 (13.77)	13.38 (13.21–13.54)	
Midwest	50,203 (26.09)	22.83 (22.63–23.04)	
South	68,610 (35.65)	21.1 (20.94–21.26)	
West	47,141(24.49)	24.05 (23.83–24.27)	
Race/Ethnicity			P < 0.001
NH Asian or Pacific Islander	2657 (1.38)	8.96 (8.62–9.3)	
NH Black or African American	12,617 (6.56)	17. 87 (17.55–18.18)	
NH White	168,537 (87.57)	21.62 (21.52–21.72)	
Hispanic or Latino	7827 (4.07)	14.3 (13.98–14.61)	
Urbanization			P < 0.001
Metropolitan	146,872 (76.31)	19.09 (18.99–19.19)	
Nonmetropolitan	45,587 (23.69)	27.23 (26.98–27.48)	
Place of death			
Medical Facility	30,272 (15.73)	–	
Decedent's Home	36,264 (18.84)	–	
Hospice Facility	5775 (3)	–	
Nursing Home/Long-term Care Facility	108,758 (56.51)	–	
Others	10,932 (5.68)	–	
Unknown	458 (0.24)		

n = total number; AAMR = Age Adjusted Mortality Rates; CI = Confidence Interval; NH = Non Hispanic.

### 3.1 Overall and gender trends of Alzheimer's and heart failure related mortality (1999–2020)

**Fig. 1:**
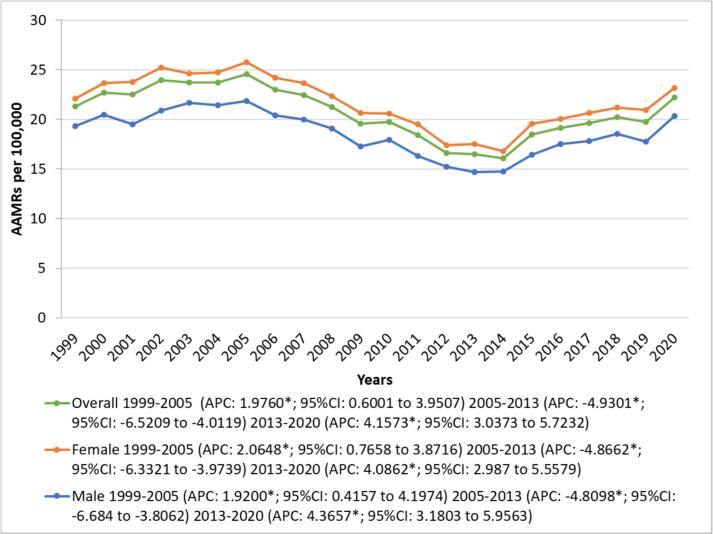
Overall and Sex-Stratified Alzheimer's disease and Heart Failure-Related AAMRs per 100,000 in Older Adults in the United States, 1999 to 2020.

Overall, the AAMR increased from 21.32 in 1999 to 24.56 in 2005 with a significant upward trend (APC: 1.9760; 95 % CI: 0.6001 to 3.9507). This was followed by a significant decline, as AAMR decreased to 16.52 in 2013 (APC: -4.9301; 95 % CI: −6.5209 to −4.0119). After 2013, the trend reversed, with AAMR rising again to 22.21 in 2020 (APC: 4.1573; 95 % CI: 3.0373 to 5.7232). (Fig. 1, Supplementary Tables 4 and 5).

During the study period, women had significantly higher total AAMRs than men [(women total AAMR: 21.57; 95 % CI: 21.46 to 21.69) vs (men total AAMR:18.41; 95 % CI: 18.26 to 18.56); *p* < 0.001]. Among men, the AAMR initially increased from 1999 to 2005 (APC: 1.9200; 95 % CI: 0.4157 to 4.1974), followed by a significant decline from 2005 to 2013 (APC: −4.8089 %; 95 % CI: −6.684 to −3.8062). However, from 2013 to 2020, the AAMR increased (APC: 4.3657; 95 % CI: 3.1803 to 5.9563). A similar trend was observed in women, with an initial increase from 1999 to 2005 (APC: 2.0648; 95 % CI: 0.7658 to 3.8716), followed by a significant decline from 2005 to 2013 (APC: -4.8662; 95 % CI: −6.3321 to −3.9739). However, from 2013 to 2020, the AAMR increased (APC: 4.0862; 95 % CI: 2.987 to 5.5579), mirroring the pattern seen in men. (Fig. 1, Supplementary Tables 4 and 5).

### 3.2 Racial trends of Alzheimer's and heart failure related mortality (1999–2020)

Among racial groups, the Non-Hispanic (NH) White (total AAMR: 21.62; 95 % CI: 21.52 to 21.72) population had the highest AAMRs followed by NH Black/African American (total AAMR: 17.87; 95 % CI: 17.55 to 18.18), Hispanic/Latino (total AAMR: 14.30; 95 % CI: 13.98 to 14.61) and NH Asian/Pacific Islander (total AAMR: 8.96; 95 % CI: 8.62 to 9.30) with pairwise comparisons between all races being statistically significant (*p* < 0.001).

**Fig. 2:**
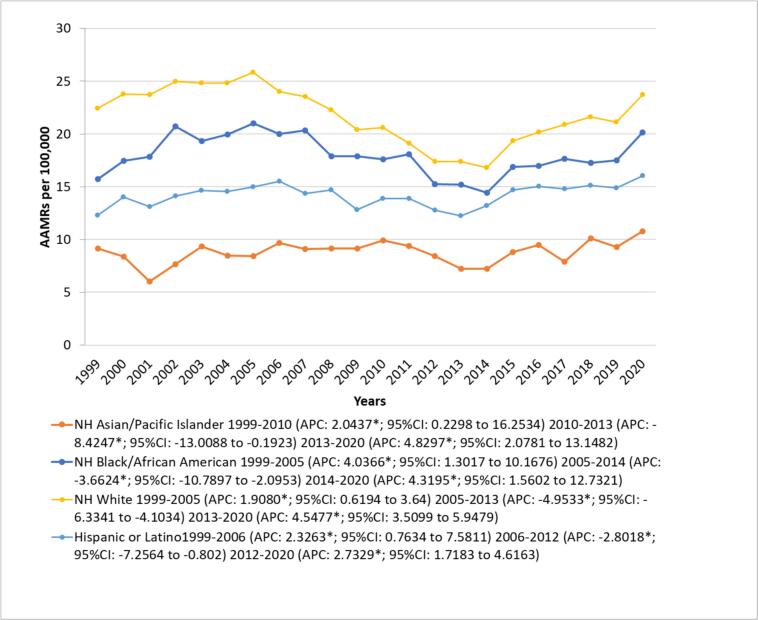
Alzheimer's disease and Heart Failure -Related AAMRs per 100,000 Stratified by Race in Older Adults in the United States, 1999 to 2020.

Among NH White individuals, AAMRs showed a notable rise from 1999 to 2005 (APC: 1.9080; 95 % CI: 0.6194 to 3.63), followed by a pronounced and statistically significant decline until 2013 (APC: -4.3195; 95 % CI: −6.3341 to −4.5477). Subsequently, a marked upward shift was observed from 2013 to 2020 (APC: 4.5477; 95 % CI: 3.5099 to 5.9479). Among NH Black/African American individuals, AAMRs exhibited a significant growth trajectory from 1999 to 2005 (APC: 4.0366; 95 % CI: 1.3017 to 10.1676), followed by a sharp and substantial decline extending to 2014 (APC: -3.6624; 95 % CI: −10.7897 to −2.0953). Thereafter, rates demonstrated another significant increase through 2020 (APC: 4.3195; 95 % CI: 1.5602 to 12.7321). NH Asian/Pacific Islander individuals experienced an initial significant rise in AAMRs from 1999 to 2010 (APC: 2.0437; 95 % CI: 0.2298 to 16.2534), followed by a considerable drop from 2010 to 2013 (APC: -8.4247; 95 % CI: −13.0088 to −0.1923). However, this downward trend reversed, with AAMRs increasing again from 2013 to 2020 (APC: 4.8297; 95 % CI: 2.0781 to 13.1482). For Hispanic/Latino individuals, AAMRs exhibited an upward trajectory until 2006 (APC: 2.3263; 95 % CI: 0.7634 to 7.5911), followed by a statistically significant decline from 2006 to 2012 (APC: -2.8018; 95 % CI: −7.2564 to −0.802). This was succeeded by a upward trend from 2012 to 2020 (APC: 2.7329; 95 % CI: 1.7183 to 4.6163). (Fig. 2, Supplemental Tables 4 and 6).

### 3.3 Geographic trends of Alzheimer's and heart failure related mortality (1999–2020)

**Fig. 3:**
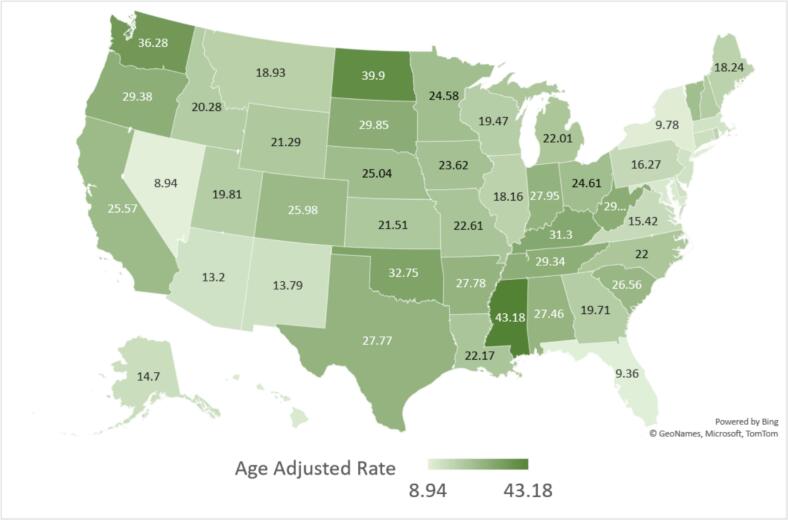
Alzheimer's disease and Heart Failure -Related AAMRs per 100,000 Stratified by State in Older Adults in the United States, 1999 to 2020.

**Fig. 4:**
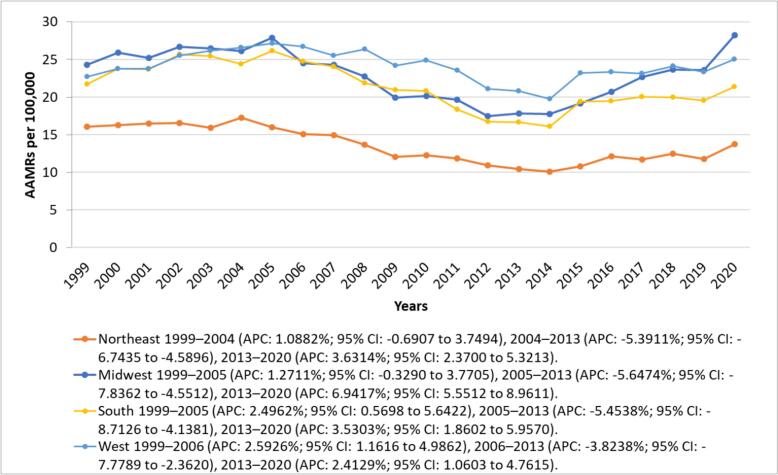
Alzheimer's disease and Heart Failure -Related AAMRs per 100,000 Stratified by Census Region in Older Adults in the United States, 1999 to 2020.

States in the 90th percentile, including Kentucky, Oklahoma, Washington, North Dakota, and Mississippi, exhibited AAMRs that were three times higher compared to those in the 10th percentile, such as Nevada, Florida, New York, the District of Columbia, and Hawaii. AAMRs varied across census regions. (Fig. 3 Supplemental Table 7). The Western region had an AAMR of 24.05 (95 % CI: 23.83 to 24.27), while the Midwestern region reported 22.83 (95 % CI: 22.63 to 23.04). In the Southern region, the AAMR was 21.1 (95 % CI: 20.94 to 21.26), whereas the Northeastern region recorded the lowest rate at 13.38 (95 % CI: 13.21 to 13.54). (Fig. 4, Supplemental table 4 and 8) Comparisons between all census regions show statistically significant differences (*p* < 0.001).

**Fig. 5:**
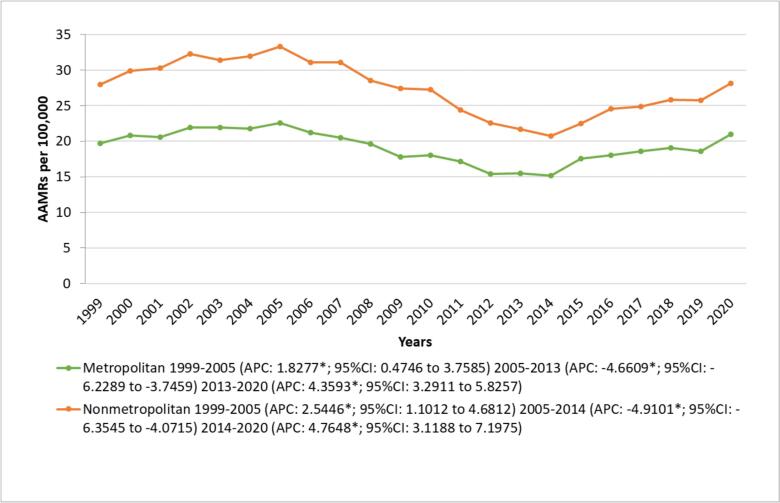
Alzheimer's disease and Heart Failure -Related AAMRs per 100,000 Stratified by Urbanization Older Adults in in the United States, 1999 to 2020.

Throughout the study period, nonmetropolitan areas had significantly higher total AAMRs compared to metropolitan areas [(nonmetropolitan total AAMR: 27.23; 95 % CI: 26.98 to 27.48) vs (metropolitan total AAMR: 19.09; 95 % CI: 18.99 to 19.19); p < 0.001]. Both areas initially experienced a significant increase in AAMRs from 1999 to 2005 (metropolitan APC: 1.8277; 95 % CI: 0.4746 to 3.7585; non-metropolitan APC: 2.5446; 95 % CI: 1.1012 to 4.6812). This trend was followed by decrease in AAMRs for metropolitan areas from 2005 to 2013 (APC: -4.6609; 95 % CI: −6.2289 to −3.7459) and non-metropolitan areas from 2005 to 2014 (APC: -4.9101; 95 % CI: −6.3535 to −4.0715). Subsequently, a marked upward shift was observed for both the groups up to 2020 (metropolitan APC: 4.3593; 95 % CI: 3.2911 to 5.8257; non-metropolitan APC: 4.7648; 95 % CI: 3.1188 to 7.1975). (Fig. 5, Supplemental Tables 4 and 9).

### 3.4 Underlying cause of death in Alzheimer's and heart failure related mortality (1999–2020)

Among patients having AD and HF listed as a cause of death on their death certificates the top 15 underlying causes of death included AD (87,393 deaths; 45.41 %), diseases of the heart (including but not limited to HF) (74,356; 38.65 %), chronic lower respiratory diseases (5682; 2.95 %), diabetes mellitus (3397; 1.76 %), cerebrovascular diseases (3214; 1.67 %), malignant neoplasms (3098; 1.61 %), influenza and pneumonia (2219; 1.15 %), accidents (unintentional injuries) (1351; 0.70 %), nephritis, nephrotic syndrome, and nephrosis (1269; 0.66 %), hypertensive diseases (988; 0.51 %), pneumonitis due to solids and liquids (884; 0.46 %), COVID-19 (750; 0.39 %), atherosclerosis (614; 0.32 %), septicemia (580; 0.30 %), and Parkinson's disease (389; 0.20 %). AD was the leading underlying cause of death, accounting for the highest proportional mortality and reporting the highest AAMR at 9.32 (95 % CI: 9.26 to 9.39), being followed by diseases of the heart with a total AAMR of 7.91 (95 % CI: 7.85 to 7.96) and Chronic lower respiratory diseases at 0.62 (95 % CI: 0.60 to 0.64). An extensive overview of the underlying causes of deaths is reported in Supplementary Table 10.

### 3.5 Alzheimer's and ischemic cardiomyopathy related mortality (1999–2020)

AD and ICM were responsible for a total of 8425 deaths between 1999 and 2020. The highest number of deaths took place in nursing homes (*n* = 4124; 48.96) followed by decedents' homes (*n* = 1799; 21.36), medical facilities (*n* = 1436; 17.04), and hospice facilities (*n* = 495; 5.88). A total of 556 (6.60) deaths occurred at other locations, while data for 15 (0.18) deaths were unknown. Deaths were equally distributed between genders, with males (1.21; 95 % CI: 1.18 to 1.25) having a higher total AAMR than females (0.77; 95 % CI: 0.74 to 0.79), with differences being statistically significant (*p* < 0.001). NH Whites were responsible for the greatest proportion of deaths at 88.3 % and had the highest AAMR at 0.97 (95 % CI: 0.95 to 1.00). They were followed by the Hispanic or Latino population at 0.78 (95 % CI: 0.70 to 0.85), the NH Black or African Americans at 0.63 (95 % CI: 0.57 to 0.69), and lastly the NH Asian or Pacific Islanders at 0.35 (95 % CI: 0.28 to 0.42). Pairwise comparisons between all four races were statistically significant (*p* < 0.003). Metropolitan areas (0.91; 95 % CI: 0.89 to 0.93) accounted for a higher number of deaths, but nonmetropolitan areas had a significantly higher overall AAMR (1.01; 95 % CI: 0.96 to 1.06) (*p* < 0.001). AAMRs were highest in the Midwest (1.08; 95 % CI: 1.04 to 1.13), then the West (0.97; 95 % CI: 0.93 to 1.02), South (0.91; 95 % CI: 0.88 to 0.94), and lowest in the Northeast (0.68; 95 % CI: 0.64 to 0.72). All pairwise comparisons between census regions were significant (p < 0.001) except West vs South (*p* = 0.052). An overview of this data is provided in Supplementary Table 11.

## 4 Discussion

Our comprehensive analysis of AD and HF-related mortality from 1999 to 2020 reveals significant epidemiological patterns, underscoring the growing burden of these comorbid conditions on public health. AD and HF frequently coexist, amplifying the risk of adverse outcomes, including increased hospitalizations and mortality [16]. Prior research has established a bidirectional relationship between cognitive decline and cardiovascular disease, with HF contributing to cerebral hypoperfusion and neuroinflammation, which accelerate AD progression [17]. Conversely, AD-related neuropathological changes, such as β-amyloid deposition, have been linked to myocardial dysfunction and worsening HF outcomes [18]. National data indicate that individuals with both conditions have a two- to threefold higher mortality risk compared to those with either condition alone [9]. Despite growing recognition, mortality trends in patients with coexisting AD and HF remain underexplored. Our study highlights time-related and demographic disparities in AAMRs, urging targeted public health interventions.

Our analysis of AAMRs due to AD and HF-related mortality revealed significant temporal fluctuations from 1999 to 2020. Initially, AAMRs exhibited a notable increase from 1999 to 2005, followed by a substantial decline until 2013. However, after 2013, this downward trend reversed, with AAMRs rising again through 2020. These patterns align with broader mortality trends observed in HF and neurodegenerative diseases, where improvements in treatment and management initially led to reductions in mortality [19], followed by subsequent increases due to the aging population and disease burden [9]. Advancements in HF management, including guideline-directed medical therapy (GDMT) and cardiovascular interventions, may have reduced mortality risk in AD-HF patients. Research from the Framingham Heart Study [20] and the Cardiovascular Health Study [21] suggests that improvements in cardiovascular care contributed to a decline in dementia incidence from the late 1970s to 2010.

The sharp rise in AAMRs after 2013 highlights a multifactorial phenomenon driven by AD's growing prevalence, an aging population with increased life expectancy, rising comorbidities, and healthcare disparities [22]. The aging population remains a primary contributor, as the proportion of adults aged 65 and older has grown significantly in the U.S. over the past decade [23], increasing the overall burden of age-related diseases such as AD and HF [24]. Additionally, metabolic risk factors such as diabetes, obesity, and hypertension have surged in recent years, contributing to both HF progression and cognitive decline [25]. Environmental and lifestyle factors may have further compounded this trend. Sedentary behavior, poor dietary habits, and surge of increasing air pollution exposure in the recent decade has been linked to both cardiovascular and neurodegenerative diseases [26]. Collectively, these factors underscore the need for enhanced prevention strategies and targeted healthcare interventions to mitigate the rising burden of AD and HF mortality.

Gender disparities in AD and HF-related mortality were evident throughout the study period, with women exhibiting consistently higher AAMRs than men. Women's longer life expectancy increases their susceptibility to age-related diseases like AD and HF. While aging is the primary risk factor for AD, the higher prevalence in women persists even after adjusting for survival differences [27,28]. Additionally, AD disproportionately affects women, who account for nearly two-thirds of all cases, partly due to genetic factors such as the APOE-ε4 allele, which has a stronger association with AD in women [29], and the neuroprotective role of estrogen, which declines after menopause, leading to increased neuroinflammation and β-amyloid accumulation [30]. Women also exhibit a higher prevalence of HF with preserved ejection fraction (HFpEF), a subtype of HF strongly associated with systemic inflammation, endothelial dysfunction, and vascular cognitive impairment, all of which contribute to increased mortality in those with coexisting AD [31]. Furthermore, studies suggest that women with HF are less likely to receive GDMT or advanced interventions such as implantable devices, potentially worsening cardiovascular outcomes and increasing mortality risk [32]. Additionally, sociocultural factors may contribute to the observed disparities, as older women are more likely to be widowed, experience social isolation, and face challenges in managing their medical conditions, leading to increased mortality [33]. These findings highlight the need for sex-specific approaches in HF management, improved AD prevention strategies, and targeted interventions to reduce the burden of these conditions in aging women.

Racial disparities in AD and HF mortality were evident in our study, with NH White individuals experiencing the highest AAMRs, followed by NH Black/African American, Hispanic/Latino, and NH Asian/Pacific Islander populations. The higher mortality burden among NH White individuals may be attributed to a combination of aging demographics and lifestyle factors, as they have a larger proportion of older adults who are more susceptible to both AD and HF [15]. NH Black/African American individuals had the second-highest AAMRs, aligning with prior research showing higher burdens of hypertension, diabetes, and chronic kidney disease—key HF risk factors—within this group [34]. Structural healthcare disparities, including reduced access to advanced HF treatments and lower utilization of AD-related diagnostic services, also contribute to elevated mortality [35]. Hispanic/Latino individuals showed lower AAMRs compared to NH White and NH Black populations. The “Hispanic Paradox”—where Hispanic individuals experience lower cardiovascular mortality despite higher rates of metabolic syndrome and diabetes—may explain this [36]. Stronger social support networks, healthier traditional diets, and lower smoking rates are protective factors [37]. NH Asian/Pacific Islander individuals had the lowest AAMRs, possibly due to genetic, dietary, and lifestyle factors. This group has lower obesity rates, healthier eating habits, and lower smoking prevalence, contributing to reduced cardiovascular risk [38,39]. However, some subgroups, particularly South Asians, are at higher risk for cardiovascular disease, highlighting the need for more nuanced subgroup analyses [40]. These disparities underscore the importance of tailored public health strategies and equitable access to preventive and therapeutic care to reduce AD and HF mortality across racial and ethnic groups.

The geographic heterogeneity in AD and HF-related mortality observed in this study reveals striking disparities shaped by both rural-urban status and regional location. Non-metropolitan areas consistently bore a higher mortality burden than metropolitan counterparts (27.23 vs. 19.09 per 100,000), with a more pronounced and prolonged initial increase, slower decline, and steeper post-2014 resurgence in AAMRs—likely reflecting delayed implementation of public health interventions, fewer specialty care services, and persistent structural healthcare deficits in rural settings [41]. These rural disadvantages appear to be mirrored in regional disparities, with states in the 90th percentile (e.g., Kentucky, Oklahoma, Mississippi) exhibiting AAMRs nearly threefold higher than those in the 10th percentile (e.g., New York, Florida, Hawaii), and the Western and Midwestern census regions showing considerably higher rates than the Northeast, which reported the lowest (13.38 per 100,000). The clustering of high-mortality states in the South and Midwest reflects a mix of factors, including high rates of cardiovascular risk, chronic illness, obesity, and smoking, along with limited access to care and persistent socioeconomic deprivation, especially in the South [42]. These disparities are deepened by structural issues such as poverty, poor walkability, underfunded health systems, and the lack of statewide smoking bans—contributing to shorter, less healthy lives [42,43]. In contrast, states and regions with lower mortality likely benefit from denser healthcare infrastructure, more robust preventative care programs, and greater public health investment—characteristics typical of coastal metropolitan centers [44]. The steeper post-2014 rise in mortality, particularly in non-metropolitan regions, underscores the growing vulnerability of these populations in the face of an aging demographic and calls for geographically tailored interventions to ensure equitable care delivery and outcome improvement.

### 4.1 Limitations

Our study has several limitations. The reliance on ICD-10 codes and death certificate data from the CDC WONDER database introduces a risk of misclassification or underreporting of Alzheimer's disease and heart failure-related deaths, potentially affecting mortality estimates. Moreover, a lack of validation data for ICD-10 codes exists in CDC WONDER. Some studies have evaluated the positive predictive values of AD ICD-10 codes while assessing mortality in other databases, as nearly 50 % (95 % CI: 6.80 to 93.2); however, these findings are not generalizable. [45] Consequently, diagnostic misclassification and inaccuracies in mortality coding remain possible. The database lacks detailed clinical variables such as disease severity, comorbid conditions, treatment history, or cognitive and functional status, limiting a more comprehensive understanding of patient outcomes. More specifically, the database lacks data on left ventricular ejection fraction and ICD-10 codes necessary to classify HF into preserved or reduced ejection fraction subtypes. Additionally, procedural data such as implantation of an automatic implantable cardioverter-defibrillator, all of which could have major implications on disease and patient outcomes. Additionally, key socioeconomic factors like income, education, healthcare access, and insurance status are not captured, restricting insights into disparities in disease burden and healthcare utilization. The absence of information on diagnostic methods, including imaging and biomarker use, may contribute to variability in case identification AD can only be confirmed histologically at autopsy; however, a clinical diagnosis is primarily used as a criterion of listing AD in death certificates. This introduces a greater risk of misdiagnosis and physician reporting errors in our results. Moreover, our analysis lacks data on certain races, especially NH American Indian/Alaskan Natives, due to unreliable data available from CDC WONDER. Furthermore, differences in healthcare infrastructure and reporting practices across states and rural versus urban settings could influence mortality trends. Lastly, the use of aggregate data prevents the exploration of causal relationships between mortality trends and healthcare interventions, requiring further studies with individual-level data for a more nuanced analysis.

## 5 Conclusion

Alzheimer's disease mortality in individuals with coexisting heart failure has demonstrated significant temporal and demographic variations from 1999 to 2020. Our findings highlight an initial rise in AAMRs, followed by a decline until 2013, and a subsequent resurgence, underscoring the growing burden of these comorbid conditions. Women, non-Hispanic White individuals, residents of the Western region, and those in nonmetropolitan areas exhibited the highest mortality rates. Targeted interventions, better healthcare access, and integrated management are key to reducing disparities. A multidisciplinary approach is crucial for improving outcomes. These findings should be interpreted with caution due to the limited clinical and socioeconomic data available in the CDC WONDER database, which may impact the analyses and influence the precision, generalizability, and overall conclusions drawn from this study.

## Clinical trial registry

Not applicable.

## Informed consent to participate

As the CDC WONDER database lacks patient and hospital identifiers, informed consent was not required for this study and was waived by human subject determination on behalf of IRB committee.

## Funding acknowledgement

No funding was used to conduct the study.

## CRediT authorship contribution statement

**Yasmeen Shaikh:** Resources, Data curation, Conceptualization. **Syeda Shahnoor:** Methodology, Investigation, Formal analysis. **Muhammad Ahmed Ali Fahim:** Resources, Methodology, Investigation. **Abdul Moiz Khan:** Validation, Software, Funding acquisition. **Tasleem Shaikh:** Writing – original draft, Visualization, Resources. **Abdul Moeed:** Writing – review & editing, Software, Project administration. **Muhammad Sohaib Asghar:** Writing – review & editing, Project administration, Formal analysis, Data curation.

## Ethical approval

Ethical approval not required to conduct the study and IRB waiver was obtained from AdventHealth Research Institute IRBnet#2159563.

## Declaration of competing interest

The authors declare that they have no known competing financial interests or personal relationships that could have appeared to influence the work reported in this paper.

## Data Availability

All the datasets generated from the above resources can be made available on a reasonable request from the corresponding author. No datasets can be made publicly available on a data repository platform without taking the adequate permission levels from the CDC WONDER.

## Appendix A Supplementary data

Supplementary tables Image 1
