# Supplementary material for: Trends in Alzheimer's disease and heart failure-related mortality among older American adults: Insights from the CDC WONDER database
Source: Am Heart J Plus. 2025 Nov 14;60:100677. doi: 10.1016/j.ahjo.2025.100677 (PMC12666518; doi:10.1016/j.ahjo.2025.100677)
Supplement: Supplementary file 1 — Supplementary tables [file mmc1.docx]

Supplemental Table 1 Alzheimer’s disease and heart failure-related Deaths, Stratified by Sex and Race, in Older Adults in the United States, 1999 to 2020

| **Deaths** | | | | | | | | |
| --- | --- | --- | --- | --- | --- | --- | --- | --- |
| **Year** | **Overall** | **Women** | **Men** | **NH White** | **NH Black or African American** | **NH Asian or Pacific Islander** | **Hispanic or Latino** | **Population** |
| 1999 | 7271 | 5089 | 2182 | 6645 | 400 | 52 | 154 | 34797841 |
| 2000 | 7855 | 5514 | 2341 | 7144 | 451 | 49 | 181 | 34991753 |
| 2001 | 7935 | 5637 | 2298 | 7225 | 462 | 39 | 183 | 35290291 |
| 2002 | 8537 | 6044 | 2493 | 7708 | 536 | 53 | 202 | 35522207 |
| 2003 | 8628 | 5958 | 2670 | 7792 | 505 | 72 | 225 | 35863529 |
| 2004 | 8751 | 6062 | 2689 | 7891 | 527 | 69 | 234 | 36203319 |
| 2005 | 9288 | 6461 | 2827 | 8360 | 568 | 74 | 256 | 36649798 |
| 2006 | 8933 | 6204 | 2729 | 7970 | 552 | 92 | 284 | 37164107 |
| 2007 | 8940 | 6178 | 2762 | 7972 | 574 | 91 | 277 | 37825711 |
| 2008 | 8664 | 5954 | 2710 | 7695 | 521 | 98 | 301 | 38777621 |
| 2009 | 8142 | 5609 | 2533 | 7178 | 540 | 105 | 280 | 39623175 |
| 2010 | 8374 | 5681 | 2693 | 7356 | 545 | 120 | 320 | 40267984 |
| 2011 | 8134 | 5582 | 2552 | 7057 | 579 | 125 | 351 | 41394141 |
| 2012 | 7495 | 5038 | 2457 | 6478 | 511 | 122 | 348 | 43145356 |
| 2013 | 7622 | 5170 | 2452 | 6593 | 526 | 115 | 355 | 44704074 |
| 2014 | 7575 | 5041 | 2534 | 6487 | 514 | 125 | 412 | 46243211 |
| 2015 | 8890 | 5969 | 2921 | 7545 | 628 | 166 | 491 | 47760852 |
| 2016 | 9395 | 6218 | 3177 | 7968 | 652 | 189 | 530 | 49244195 |
| 2017 | 9821 | 6478 | 3343 | 8352 | 699 | 170 | 557 | 50858679 |
| 2018 | 10306 | 6742 | 3564 | 8728 | 708 | 228 | 597 | 52431193 |
| 2019 | 10227 | 6723 | 3504 | 8621 | 739 | 226 | 603 | 54058263 |
| 2020 | 11676 | 7561 | 4115 | 9772 | 880 | 277 | 686 | 55659365 |
| **Total** | **192459** | **130913** | **61546** | **168,537** | **12,617** | **2,657** | **7,827** | **928476665** |

NH, non-Hispanic.

Supplemental Table 2 Alzheimer’s disease and heart failure-related Deaths, Stratified by Autopsy status, in Older Adults in the United States, 1999 to 2020

| Year | No autopsy performed | Autopsy performed | Unknown | Total |
| --- | --- | --- | --- | --- |
| 1999 | – | – | 7,271 | 7,271 |
| 2000 | – | – | 7,855 | 7,855 |
| 2001 | – | – | 7,935 | 7,935 |
| 2002 | – | – | 8,537 | 8,537 |
| 2003 | 7,184 | 39 | 1,405 | 8,628 |
| 2004 | 7,261 | 37 | 1,453 | 8,751 |
| 2005 | 8,276 | 37 | 975 | 9,288 |
| 2006 | 8,013 | 32 | 888 | 8,933 |
| 2007 | 8,053 | 36 | 851 | 8,940 |
| 2008 | 7,845 | 32 | 787 | 8,664 |
| 2009 | 7,392 | 28 | 722 | 8,142 |
| 2010 | 7,605 | 24 | 745 | 8,374 |
| 2011 | 7,463 | 18 | 653 | 8,134 |
| 2012 | 7,119 | 17 | 359 | 7,495 |
| 2013 | 7,269 | 11 | 342 | 7,622 |
| 2014 | 7,176 | 15 | 384 | 7,575 |
| 2015 | 8,339 | 17 | 534 | 8,890 |
| 2016 | 9,111 | 13 | 271 | 9,395 |
| 2017 | 9,593 | 10 | 218 | 9,821 |
| 2018 | 10,030 | 24 | 252 | 10,306 |
| 2019 | 9,926 | 34 | 267 | 10,227 |
| 2020 | 11,369 | 40 | 267 | 11,676 |
| Total | 142,359 | 495 | 33,320 | 176,174 |

**Supplemental Table 3 Alzheimer’s disease and heart failure-related** Mortality, Stratified by Place of Death in Older Adults in the United States, 1999 to 2020

| **Deaths** | | | | | |
| --- | --- | --- | --- | --- | --- |
| **Year** | **Medical Facility** | **Nursing Home** | **Hospices** | **Home** | **Other** |
|  |  |  |  |  |  |
| 1999 | 1801 | 4436 | - | 837 | 196 |
| 2000 | 1797 | 4849 | - | 927 | 279 |
| 2001 | 1770 | 4917 | - | 945 | 300 |
| 2002 | 1906 | 5188 | - | 1065 | 376 |
| 2003 | 1848 | 5197 | 11 | 1170 | 384 |
| 2004 | 1832 | 5214 | 23 | 1251 | 421 |
| 2005 | 1825 | 5565 | 86 | 1370 | 427 |
| 2006 | 1675 | 5284 | 81 | 1447 | 399 |
| 2007 | 1583 | 5270 | 136 | 1489 | 415 |
| 2008 | 1516 | 5043 | 167 | 1415 | 407 |
| 2009 | 1309 | 4538 | 187 | 1486 | 448 |
| 2010 | 1308 | 4833 | 205 | 1534 | 492 |
| 2011 | 1213 | 4657 | 232 | 1576 | 456 |
| 2012 | 1096 | 4280 | 221 | 1462 | 435 |
| 2013 | 1016 | 4254 | 309 | 1524 | 516 |
| 2014 | 874 | 4156 | 377 | 1706 | 460 |
| 2015 | 940 | 4758 | 544 | 2053 | 590 |
| 2016 | 961 | 4925 | 570 | 2206 | 731 |
| 2017 | 945 | 5116 | 651 | 2344 | 763 |
| 2018 | 1005 | 5256 | 648 | 2605 | 790 |
| 2019 | 990 | 5254 | 653 | 2552 | 776 |
| 2020 | 1062 | 5768 | 674 | 3300 | 871 |
| **Total** | **30272** | **108758** | **5775** | **36264** | **10932** |

**Supplemental Table 4** Annual percent change (APC) of Alzheimer’s disease and heart failure-related Age-Adjusted Mortality Rates per 100,000 in Older Adults in the United States, 1999 to 2020

| **Year Interval** | **APC (95% CI)** |
| --- | --- |
| **Overall** | |
| 1999-2005 | 1.9760*(0.6001 to 3.9507) |
| 2005-2013 | -4.9301*(-6.5209 to -4.0119) |
| 2013-2020 | 4.1573*(3.0373 to 5.7232) |
| **Male** | |
| 1999-2005 | 1.9200*(0.4157 to 4.1974) |
| 2005-2013 | -4.8098*(-6.684 to -3.8062) |
| 2013-2020 | 4.3657*(3.1803 to 5.9563) |
| **Female** | |
| 1999-2005 | 2.0648*(0.7658 to 3.8716) |
| 2005-2013 | -4.8662*(-6.3321 to -3.9739) |
| 2013-2020 | 4.0862*(2.987 to 5.5579) |
| **NH White** | |
| 1999-2005 | 1.9080*(0.6194 to 3.64) |
| 2005-2013 | -4.9533*(-6.3341 to -4.1034) |
| 2013-2020 | 4.5477*(3.5099 to 5.9479) |
| **NH Black or African American** | |
| 1999-2005 | 4.0366*(1.3017 to 10.1676) |
| 2005-2014 | -3.6624*(-10.7897 to -2.0953) |
| 2014-2020 | 4.3195*(1.5602 to 12.7321) |
| **NH Asian or Pacific Islander** | |
| 1999-2010 | 2.0437*(0.2298 to 16.2534) |
| 2010-2013 | -8.4247*(-13.0088 to -0.1923) |
| 2013-2020 | 4.8297*(2.0781 to 13.1482) |
| **Hispanic or Latino** | |
| 1999-2006 | 2.3263*(0.7634 to 7.5811) |
| 2006-2012 | -2.8018*(-7.2564 to -0.802) |
| 2012-2020 | 2.7329*(1.7183 to 4.6163) |
| **Metropolitan areas** | |
| 1999-2005 | 1.8277*(0.4746 to 3.7585) |
| 2005-2013 | -4.6609*(-6.2289 to -3.7459) |
| 2013-2020 | 4.3593*(3.2911 to 5.8257) |
| **Nonmetropolitan area** | |
| 1999-2005 | 2.5446*(1.1012 to 4.6812) |
| 2005-2014 | -4.9101*(-6.3545 to -4.0715) |
| 2014-2020 | 4.7648*(3.1188 to 7.1975) |
| **Northeast region** | |
| 1999-2004 | 1.0882(-0.6907 to 3.7494) |
| 2004-2013 | -5.3911*(-6.7435 to -4.5896) |
| 2013-2020 | 3.6314*(2.37 to 5.3213) |
| **Midwest region** | |
| 1999-2005 | 1.2711(-0.329 to 3.7705) |
| 2005-2013 | -5.6474*(-7.8362 to -4.5512) |
| 2013-2020 | 6.9417*(5.5512 to 8.9611) |
| **South region** | |
| 1999-2005 | 2.4962*(0.5698 to 5.6422) |
| 2005-2013 | -5.4538*(-8.7126 to -4.1381) |
| 2013-2020 | 3.5303*(1.8602 to 5.957) |
| **West region** | |
| 1999-2006 | 2.5926*(1.1616 to 4.9862) |
| 2006-2013 | -3.8238*(-7.7789 to -2.362) |
| 2013-2020 | 2.4129*(1.0603 to 4.7615) |

APC = annual percent change; NH = non-Hispanic; * Indicates that the annual percentage change (APC) is significantly different from zero at α = 0.05. AAMR = age-adjusted mortality rate.

**Supplemental Table 5** Overall and Sex‐Stratified Alzheimer’s disease and heart failure-related Age-Adjusted Mortality Rates per 100,000 in Older Adults in the United States, 1999 to 2020

| **Age-Adjusted Rate (95% CI)** | | | |
| --- | --- | --- | --- |
| **Year** | **Male** | **Female** | **Overall** |
| 1999 | 19.32 (18.5–20.15) | 22.11 (21.5–22.72) | 21.32 (20.83–21.81) |
| 2000 | 20.46 (19.62–21.3) | 23.65 (23.03–24.28) | 22.68 (22.18–23.18) |
| 2001 | 19.53 (18.72–20.34) | 23.78 (23.16–24.4) | 22.53 (22.04–23.03) |
| 2002 | 20.89 (20.06–21.72) | 25.23 (24.59–25.87) | 23.94 (23.43–24.44) |
| 2003 | 21.69 (20.86–22.52) | 24.61 (23.99–25.24) | 23.74 (23.24–24.24) |
| 2004 | 21.45 (20.63–22.27) | 24.73 (24.11–25.35) | 23.73 (23.23–24.23) |
| 2005 | 21.88 (21.07–22.7) | 25.79 (25.16–26.42) | 24.56 (24.07–25.06) |
| 2006 | 20.43 (19.66–21.2) | 24.18 (23.58–24.79) | 23.02 (22.54–23.49) |
| 2007 | 20.00 (19.25–20.74) | 23.64 (23.05–24.24) | 22.47 (22.01–22.94) |
| 2008 | 19.09 (18.37–19.81) | 22.32 (21.75–22.89) | 21.28 (20.83–21.72) |
| 2009 | 17.27 (16.6–17.95) | 20.65 (20.1–21.19) | 19.56 (19.13–19.99) |
| 2010 | 17.97 (17.29–18.65) | 20.6 (20.05–21.14) | 19.72 (19.3–20.15) |
| 2011 | 16.29 (15.66–16.93) | 19.5 (18.98–20.02) | 18.42 (18.02–18.83) |
| 2012 | 15.23 (14.63–15.84) | 17.42 (16.93–17.91) | 16.63 (16.25–17.01) |
| 2013 | 14.68 (14.1–15.26) | 17.53 (17.05–18.02) | 16.52 (16.15–16.9) |
| 2014 | 14.74 (14.16–15.31) | 16.82 (16.35–17.29) | 16.07 (15.71–16.44) |
| 2015 | 16.42 (15.82–17.01) | 19.56 (19.05–20.06) | 18.49 (18.1–18.88) |
| 2016 | 17.51 (16.9–18.12) | 20.07 (19.56–20.58) | 19.15 (18.76–19.54) |
| 2017 | 17.84 (17.23–18.45) | 20.64 (20.13–21.15) | 19.61 (19.22–20.00) |
| 2018 | 18.55 (17.94–19.16) | 21.2 (20.69–21.71) | 20.24 (19.85–20.64) |
| 2019 | 17.78 (17.19–18.37) | 20.98 (20.47–21.48) | 19.77 (19.39–20.16) |
| 2020 | 20.36 (19.74–20.99) | 23.2 (22.68–23.73) | 22.21 (21.8–22.61) |
| **Total** | **18.41 (18.26–18.56)** | **21.57 (21.46–21.69)** | **20.56 (20.47–20.65)** |

Supplemental Table 6 Race‐Stratified Alzheimer’s disease and heart failure-related Age-Adjusted Mortality Rates per 100,000 in Older Adults in the United States, 1999 to 2020

| **Age-Adjusted Rate (95% CI)** | | | | |
| --- | --- | --- | --- | --- |
| **Year** | **NH White** | **NH Black or African American** | **Hispanic or Latino** | **NH Asian or Pacific Islander** |
| 1999 | 22.44 (21.90–22.98) | 15.72 (14.18–17.26) | 12.29 (10.33–14.24) | 9.13 (6.77–12.03) |
| 2000 | 23.76 (23.21–24.31) | 17.48 (15.87–19.10) | 14.00 (11.95–16.04) | 8.38 (6.18–11.11) |
| 2001 | 23.71 (23.17–24.26) | 17.85 (16.22–19.48) | 13.13 (11.21–15.04) | 6.03 (4.27–8.28) |
| 2002 | 24.95 (24.40–25.51) | 20.71 (18.95–22.46) | 14.10 (12.15–16.06) | 7.64 (5.71–10.02) |
| 2003 | 24.85 (24.30–25.40) | 19.31 (17.62–21.00) | 14.63 (12.70–16.56) | 9.35 (7.29–11.81) |
| 2004 | 24.85 (24.30–25.40) | 19.95 (18.24–21.65) | 14.54 (12.66–16.42) | 8.48 (6.58–10.75) |
| 2005 | 25.82 (25.27–26.37) | 20.99 (19.26–22.71) | 14.98 (13.13–16.83) | 8.45 (6.62–10.62) |
| 2006 | 24.00 (23.47–24.52) | 19.99 (18.32–21.66) | 15.49 (13.67–17.30) | 9.68 (7.80–11.89) |
| 2007 | 23.52 (23.00–24.04) | 20.34 (18.67–22.01) | 14.36 (12.67–16.06) | 9.08 (7.31–11.15) |
| 2008 | 22.30 (21.80–22.80) | 17.91 (16.37–19.45) | 14.71 (13.04–16.38) | 9.17 (7.43–11.18) |
| 2009 | 20.41 (19.93–20.88) | 17.88 (16.37–19.39) | 12.83 (11.32–14.33) | 9.13 (7.38–10.88) |
| 2010 | 20.58 (20.11–21.06) | 17.61 (16.13–19.10) | 13.90 (12.37–15.43) | 9.94 (8.16–11.72) |
| 2011 | 19.11 (18.66–19.55) | 18.09 (16.62–19.57) | 13.87 (12.42–15.33) | 9.37 (7.72–11.01) |
| 2012 | 17.38 (16.96–17.81) | 15.25 (13.92–16.57) | 12.78 (11.43–14.13) | 8.44 (6.94–9.94) |
| 2013 | 17.40 (16.98–17.82) | 15.20 (13.90–16.50) | 12.24 (10.96–13.51) | 7.23 (5.91–8.56) |
| 2014 | 16.82 (16.40–17.23) | 14.45 (13.20–15.70) | 13.20 (11.93–14.48) | 7.25 (5.98–8.52) |
| 2015 | 19.37 (18.93–19.81) | 16.88 (15.56–18.21) | 14.68 (13.38–15.98) | 8.82 (7.47–10.17) |
| 2016 | 20.17 (19.73–20.62) | 17.00 (15.69–18.31) | 15.02 (13.74–16.30) | 9.48 (8.13–10.84) |
| 2017 | 20.90 (20.45–21.36) | 17.63 (16.31–18.94) | 14.77 (13.54–16.00) | 7.92 (6.73–9.12) |
| 2018 | 21.61 (21.15–22.06) | 17.25 (15.97–18.53) | 15.11 (13.89–16.32) | 10.09 (8.78–11.41) |
| 2019 | 21.13 (20.69–21.58) | 17.49 (16.23–18.76) | 14.90 (13.71–16.09) | 9.29 (8.08–10.51) |
| 2020 | 23.74 (23.27–24.22) | 20.17 (18.83–21.51) | 16.03 (14.83–17.24) | 10.78 (9.51–12.06) |
| **Total** | **21.62 (21.52–21.72)** | **17.87 (17.55–18.18)** | **14.30 (13.98–14.61)** | **8.96 (8.62–9.30)** |

NH = non-Hispanic.

Supplemental Table 7 Alzheimer’s disease and heart failure-related Age-Adjusted Mortality Rates per 100,000, Stratified by States in Older Adults in the United States, 1999 to 2020

| **State** | **Age-Adjusted Rate (95% CI)** |
| --- | --- |
| Alabama | 27.46 (26.58–28.33) |
| Alaska | 14.70 (12.20–17.19) |
| Arizona | 13.20 (12.68–13.72) |
| Arkansas | 27.78 (26.69–28.86) |
| California | 25.57 (25.26–25.88) |
| Colorado | 25.98 (25.07–26.89) |
| Connecticut | 14.27 (13.62–14.91) |
| Delaware | 13.64 (12.26–15.03) |
| District of Columbia | 10.37 (8.84–11.89) |
| Florida | 9.36 (9.15–9.58) |
| Georgia | 19.71 (19.11–20.32) |
| Hawaii | 11.00 (10.08–11.92) |
| Idaho | 20.28 (18.93–21.64) |
| Illinois | 18.16 (17.74–18.59) |
| Indiana | 27.95 (27.20–28.69) |
| Iowa | 23.62 (22.74–24.49) |
| Kansas | 21.51 (20.59–22.44) |
| Kentucky | 31.30 (30.31–32.29) |
| Louisiana | 22.17 (21.33–23.01) |
| Maine | 18.24 (17.05–19.44) |
| Maryland | 11.33 (10.81–11.84) |
| Massachusetts | 12.95 (12.49–13.41) |
| Michigan | 22.01 (21.50–22.53) |
| Minnesota | 24.58 (23.85–25.32) |
| Mississippi | 43.18 (41.75–44.60) |
| Missouri | 22.61 (21.95–23.28) |
| Montana | 18.93 (17.46–20.41) |
| Nebraska | 25.04 (23.81–26.27) |
| Nevada | 8.94 (8.17–9.72) |
| New Hampshire | 20.40 (19.03–21.77) |
| New Jersey | 13.22 (12.80–13.63) |
| New Mexico | 13.79 (12.82–14.75) |
| New York | 9.78 (9.54–10.02) |
| North Carolina | 22.00 (21.43–22.57) |
| North Dakota | 39.90 (37.52–42.28) |
| Ohio | 24.61 (24.11–25.10) |
| Oklahoma | 32.75 (31.68–33.81) |
| Oregon | 29.38 (28.43–30.32) |
| Pennsylvania | 16.27 (15.91–16.62) |
| Rhode Island | 18.05 (16.76–19.34) |
| South Carolina | 26.56 (25.66–27.46) |
| South Dakota | 29.85 (27.93–31.78) |
| Tennessee | 29.34 (28.55–30.14) |
| Texas | 27.77 (27.33–28.21) |
| Utah | 19.81 (18.62–21.01) |
| Vermont | 24.61 (22.49–26.72) |
| Virginia | 15.42 (14.89–15.95) |
| Washington | 36.28 (35.43–37.13) |
| West Virginia | 29.93 (28.58–31.27) |
| Wisconsin | 19.47 (18.85–20.10) |
| Wyoming | 21.29 (18.97–23.61) |

Supplemental Table 8 Alzheimer’s disease and heart failure-related Age-Adjusted Mortality Rates per 100,000, Stratified by Census Region in Older Adults in the United States, 1999 to 2020

| **Census Region** | **Year** | **Age-Adjusted Rate (95% CI)** |
| --- | --- | --- |
| Northeast | 1999 | 16.06 (15.15–16.97) |
| Northeast | 2000 | 16.28 (15.38–17.19) |
| Northeast | 2001 | 16.52 (15.61–17.43) |
| Northeast | 2002 | 16.56 (15.66–17.46) |
| Northeast | 2003 | 15.93 (15.05–16.81) |
| Northeast | 2004 | 17.26 (16.35–18.16) |
| Northeast | 2005 | 16.01 (15.14–16.87) |
| Northeast | 2006 | 15.10 (14.27–15.93) |
| Northeast | 2007 | 14.93 (14.12–15.75) |
| Northeast | 2008 | 13.67 (12.89–14.44) |
| Northeast | 2009 | 12.04 (11.32–12.76) |
| Northeast | 2010 | 12.26 (11.53–12.98) |
| Northeast | 2011 | 11.84 (11.14–12.54) |
| Northeast | 2012 | 10.90 (10.22–11.57) |
| Northeast | 2013 | 10.41 (9.76–11.06) |
| Northeast | 2014 | 10.10 (9.46–10.73) |
| Northeast | 2015 | 10.82 (10.16–11.47) |
| Northeast | 2016 | 12.11 (11.42–12.80) |
| Northeast | 2017 | 11.73 (11.06–12.40) |
| Northeast | 2018 | 12.50 (11.81–13.19) |
| Northeast | 2019 | 11.79 (11.12–12.46) |
| Northeast | 2020 | 13.73 (13.01–14.45) |
| Northeast | **Total** | 13.38 (13.21–13.54) |
| Midwest | 1999 | 24.29 (23.24–25.34) |
| Midwest | 2000 | 25.90 (24.82–26.98) |
| Midwest | 2001 | 25.21 (24.16–26.27) |
| Midwest | 2002 | 26.70 (25.62–27.78) |
| Midwest | 2003 | 26.48 (25.40–27.55) |
| Midwest | 2004 | 26.15 (25.09–27.21) |
| Midwest | 2005 | 27.85 (26.77–28.94) |
| Midwest | 2006 | 24.53 (23.52–25.53) |
| Midwest | 2007 | 24.30 (23.31–25.29) |
| Midwest | 2008 | 22.74 (21.79–23.69) |
| Midwest | 2009 | 19.96 (19.08–20.85) |
| Midwest | 2010 | 20.12 (19.24–21.00) |
| Midwest | 2011 | 19.62 (18.76–20.47) |
| Midwest | 2012 | 17.50 (16.69–18.30) |
| Midwest | 2013 | 17.80 (17.00–18.61) |
| Midwest | 2014 | 17.77 (16.97–18.58) |
| Midwest | 2015 | 19.19 (18.36–20.02) |
| Midwest | 2016 | 20.74 (19.88–21.59) |
| Midwest | 2017 | 22.66 (21.77–23.55) |
| Midwest | 2018 | 23.63 (22.73–24.53) |
| Midwest | 2019 | 23.61 (22.71–24.51) |
| Midwest | 2020 | 28.21 (27.24–29.19) |
| Midwest | **Total** | 22.83 (22.63–23.04) |
| South | 1999 | 21.73 (20.88–22.57) |
| South | 2000 | 23.76 (22.88–24.64) |
| South | 2001 | 23.67 (22.79–24.54) |
| South | 2002 | 25.68 (24.77–26.58) |
| South | 2003 | 25.43 (24.54–26.32) |
| South | 2004 | 24.38 (23.51–25.25) |
| South | 2005 | 26.15 (25.26–27.03) |
| South | 2006 | 24.75 (23.90–25.60) |
| South | 2007 | 24.03 (23.20–24.86) |
| South | 2008 | 21.89 (21.11–22.67) |
| South | 2009 | 20.98 (20.22–21.73) |
| South | 2010 | 20.82 (20.07–21.56) |
| South | 2011 | 18.36 (17.68–19.05) |
| South | 2012 | 16.74 (16.10–17.39) |
| South | 2013 | 16.64 (16.01–17.28) |
| South | 2014 | 16.13 (15.52–16.75) |
| South | 2015 | 19.41 (18.75–20.08) |
| South | 2016 | 19.47 (18.81–20.13) |
| South | 2017 | 20.03 (19.37–20.69) |
| South | 2018 | 19.99 (19.34–20.64) |
| South | 2019 | 19.57 (18.94–20.21) |
| South | 2020 | 21.36 (20.70–22.01) |
| South | **Total** | 21.10 (20.94–21.26) |
| West | 1999 | 22.73 (21.58–23.89) |
| West | 2000 | 23.79 (22.62–24.96) |
| West | 2001 | 23.77 (22.62–24.92) |
| West | 2002 | 25.49 (24.31–26.67) |
| West | 2003 | 26.18 (25.00–27.36) |
| West | 2004 | 26.57 (25.39–27.74) |
| West | 2005 | 27.16 (25.99–28.33) |
| West | 2006 | 26.70 (25.56–27.84) |
| West | 2007 | 25.55 (24.45–26.65) |
| West | 2008 | 26.34 (25.24–27.43) |
| West | 2009 | 24.19 (23.15–25.23) |
| West | 2010 | 24.91 (23.87–25.95) |
| West | 2011 | 23.57 (22.58–24.56) |
| West | 2012 | 21.12 (20.20–22.05) |
| West | 2013 | 20.80 (19.89–21.71) |
| West | 2014 | 19.74 (18.87–20.61) |
| West | 2015 | 23.19 (22.27–24.12) |
| West | 2016 | 23.34 (22.42–24.26) |
| West | 2017 | 23.12 (22.21–24.03) |
| West | 2018 | 24.09 (23.17–25.00) |
| West | 2019 | 23.36 (22.47–24.25) |
| West | 2020 | 25.04 (24.13–25.95) |
| West | **Total** | 24.05 (23.83–24.27) |
| **Total** | **Total** | 20.56 (20.47–20.65) |

**Supplemental Table 9 Alzheimer’s disease and heart failure-related** Age-Adjusted Mortality Rates per 100,000, Stratified by Urban-Rural Classification in Older Adults in the United States, 1999 to 2020

| **Age-Adjusted Rate (95% CI)** | | |
| --- | --- | --- |
| **Year** | **Metropolitan** | **Nonmetropolitan** |
| 1999 | 19.68 (19.16–20.21) | 27.96 (26.70–29.22) |
| 2000 | 20.85 (20.32–21.39) | 29.92 (28.62–31.22) |
| 2001 | 20.62 (20.09–21.14) | 30.26 (28.96–31.56) |
| 2002 | 21.91 (21.37–22.46) | 32.30 (30.96–33.65) |
| 2003 | 21.94 (21.40–22.47) | 31.41 (30.10–32.73) |
| 2004 | 21.78 (21.25–22.31) | 31.94 (30.62–33.27) |
| 2005 | 22.57 (22.04–23.10) | 33.29 (31.94–34.63) |
| 2006 | 21.19 (20.68–21.70) | 31.09 (29.80–32.38) |
| 2007 | 20.52 (20.03–21.01) | 31.12 (29.84–32.41) |
| 2008 | 19.65 (19.18–20.13) | 28.53 (27.31–29.75) |
| 2009 | 17.80 (17.35–18.24) | 27.45 (26.25–28.64) |
| 2010 | 18.05 (17.61–18.50) | 27.28 (26.10–28.46) |
| 2011 | 17.15 (16.72–17.57) | 24.43 (23.32–25.53) |
| 2012 | 15.38 (14.97–15.78) | 22.59 (21.54–23.65) |
| 2013 | 15.46 (15.06–15.85) | 21.68 (20.66–22.71) |
| 2014 | 15.15 (14.76–15.54) | 20.71 (19.71–21.71) |
| 2015 | 17.59 (17.18–18.01) | 22.53 (21.50–23.56) |
| 2016 | 18.04 (17.62–18.45) | 24.57 (23.49–25.65) |
| 2017 | 18.58 (18.16–19.00) | 24.89 (23.82–25.96) |
| 2018 | 19.08 (18.66–19.49) | 25.81 (24.73–26.89) |
| 2019 | 18.57 (18.16–18.98) | 25.74 (24.67–26.82) |
| 2020 | 21.01 (20.58–21.44) | 28.18 (27.06–29.29) |
| Total | 19.09 (18.99–19.19) | 27.23 (26.98–27.48) |

Supplementary Table 10 Alzheimer’s disease and heart failure-related underlying causes of death, in Older Adults in the United States, 1999 to 2020

| **Underlying cause of death** | **ICD-10 codes** | **Deaths (n)** | **% of total deaths** | **Crude rate (95% CI)** | **Age-adjusted mortality rate (95% CI)** |
| --- | --- | --- | --- | --- | --- |
| Alzheimer disease | G30 | 87,393 | 45.41 | 9.41 (95% CI: 9.35–9.47) | 9.32 (95% CI: 9.26–9.39) |
| Diseases of heart | I00–I09, I11, I13, I20–I51 | 74,356 | 38.65 | 8.01 (95% CI: 7.95–8.07) | 7.91 (95% CI: 7.85–7.96) |
| Chronic lower respiratory diseases | J40–J47 | 5,682 | 2.95 | 0.61 (95% CI: 0.60–0.63) | 0.62 (95% CI: 0.60–0.64) |
| Diabetes mellitus | E10–E14 | 3,397 | 1.76 | 0.37 (95% CI: 0.35–0.38) | 0.34 (95% CI: 0.33–0.35) |
| Cerebrovascular diseases | I60–I69 | 3,214 | 1.67 | 0.35 (95% CI: 0.33–0.36) | 0.34 (95% CI: 0.33–0.35) |
| Malignant neoplasms | C00–C97 | 3,098 | 1.61 | 0.33 (95% CI: 0.32–0.35) | 0.30 (95% CI: 0.29–0.31) |
| Influenza and pneumonia | J09–J18 | 2,219 | 1.15 | 0.24 (95% CI: 0.23–0.25) | 0.23 (95% CI: 0.22–0.24) |
| Accidents (unintentional injuries) | V01–X59, Y85–Y86 | 1,351 | 0.7 | 0.15 (95% CI: 0.14–0.15) | 0.15 (95% CI: 0.14–0.15) |
| Nephritis, nephrotic syndrome, and nephrosis | N00–N07, N17–N19, N25–N27 | 1,269 | 0.66 | 0.14 (95% CI: 0.13–0.14) | 0.12 (95% CI: 0.11–0.13) |
| Essential hypertension and hypertensive renal disease | I10, I12, I15 | 988 | 0.51 | 0.11 (95% CI: 0.10–0.11) | 0.11 (95% CI: 0.10–0.12) |
| Pneumonitis due to solids and liquids | J69 | 884 | 0.46 | 0.10 (95% CI: 0.09–0.10) | 0.10 (95% CI: 0.09–0.10) |
| COVID-19 | U07.1 | 750 | 0.39 | 0.08 (95% CI: 0.07–0.09) | 0.10 (95% CI: 0.09–0.10) |
| Atherosclerosis | I70 | 614 | 0.32 | 0.07 (95% CI: 0.06–0.07) | 0.08 (95% CI: 0.08–0.09) |
| Septicemia | A40–A41 | 580 | 0.3 | 0.06 (95% CI: 0.06–0.07) | 0.07 (95% CI: 0.07–0.08) |
| Parkinson disease | G20–G21 | 389 | 0.2 | 0.04 (95% CI: 0.04–0.05) | 0.06 (95% CI: 0.05–0.07) |

Supplementary Table 11 Alzheimer’s disease and Ischemic Cardiomyopathy-related mortality, in Older Adults in the United States, 1999 to 2020

| **Variable** | **Deaths n (%)** | **AAMR (per 100,000)** | **P-value of pairwise comparison** |
| --- | --- | --- | --- |
| **Overall Population** | 8425 (100.00) | 0.92 (0.90 to 0.94) |  |
| **Sex** |  |  | p < 0.001 |
| Male | 4061 (48.20) | 1.21 (1.18 to 1.25) |  |
| Female | 4364 (51.80) | 0.77 (0.74 to 0.79) |  |
| **Census Region** |  |  | * |
| Northeast | 1309 (15.54) | 0.68 (0.64 to 0.72) |  |
| Midwest | 2323 (27.57) | 1.08 (1.04 to 1.13) |  |
| South | 2959 (35.13) | 0.91 (0.88 to 0.94) |  |
| West | 1834 (21.76) | 0.97 (0.93 to 1.02) |  |
| **Race/Ethnicity** |  |  | p<0.003 |
| NH Asian or Pacific Islander | 106 (1.26) | 0.35 (0.28 to 0.42) |  |
| NH Black or African American | 437 (5.19) | 0.63 (0.57 to 0.69) |  |
| NH White | 7439 (88.30) | 0.97 (0.95 to 1.00) |  |
| Hispanic or Latino | 417 (4.95) | 0.78 (0.70 to 0.85) |  |
| **Urbanization** |  |  | p < 0.001 |
| Metropolitan | 6722 (79.80) | 0.91 (0.89 to 0.93) |  |
| Nonmetropolitan | 1703 (20.20) | 1.01 (0.96 to 1.06) |  |
| **Place of Death** |  |  |  |
| Medical Facility | 1436 (17.04) | — |  |
| Decedent’s Home | 1799 (21.36) | — |  |
| Hospice Facility | 495 (5.88) | — |  |
| Nursing Home/Long-term Care Facility | 4124 (48.96) | — |  |
| Others | 556 (6.60) | — |  |
| Unknown | 15 (0.18) | — |  |

* All pairwise comparisons were significant (p < 0.05) except West vs South (p = 0.052).
